# Supplementary material for: Complexome profiling of the Chlamydomonas psb28 mutant reveals TEF5 as an early PSII assembly factor
Source: Plant Cell. 2025 Mar 17;37(6):koaf055. doi: 10.1093/plcell/koaf055 (PMC12164590; doi:10.1093/plcell/koaf055)
Supplement: koaf055_Supplementary_Data [file koaf055_supplementary_data.zip › psb28_interactive.zip!/plots/intensity/qualityPlots/peptides/BCX1_Cre12.g484000.t1.2.html]

### Cre12.g484000.t1.2

Trivial name: BCX1  
SeqCoverage [median]: 4.80  
Mass: 49.20 kDa  
  
 

| Sequence | MissedCleavage | Mass [Da] | wtIntensity | muIntensity | Score |
| --- | --- | --- | --- | --- | --- |
| TGLQDGVR | 0 | 844.44 | 2487.07 | 496.09 | 77.32 |
| TLVAGDAASSGSSR | 0 | 1277.62 | 4909.79 | 2233.29 | 103.22 |
|  |  |  | 7396.9 | 2729.4 |  |
